# The Pneumococcal Polysaccharide Capsule and Pneumolysin Differentially Affect CXCL8 and IL-6 Release from Cells of the Upper and Lower Respiratory Tract

**DOI:** 10.1371/journal.pone.0092355

**Published:** 2014-03-24

**Authors:** Eliane Küng, William R. Coward, Daniel R. Neill, Hesham A. Malak, Kathrin Mühlemann, Aras Kadioglu, Markus Hilty, Lucy J. Hathaway

**Affiliations:** 1 Institute for Infectious Diseases, University of Bern, Bern, Switzerland; 2 Graduate School for Cellular and Biomedical Sciences, University of Bern, Bern, Switzerland; 3 Nottingham Respiratory Biomedical Research Unit, Clinical Sciences Building, Nottingham City Campus, Nottingham, United Kingdom; 4 Clinical Infection, Microbiology and Immunology, Institute of Infection & Global Health, University of Liverpool, Liverpool, United Kingdom; 5 Department of Infectious Diseases, University Hospital, Bern, Switzerland; Instituto Butantan, Brazil

## Abstract

The polysaccharide capsule and pneumolysin toxin are major virulence factors of the human bacterial pathogen *Streptococcus pneumoniae*. Colonization of the nasopharynx is asymptomatic but invasion of the lungs can result in invasive pneumonia. Here we show that the capsule suppresses the release of the pro-inflammatory cytokines CXCL8 (IL-8) and IL-6 from the human pharyngeal epithelial cell line Detroit 562. Release of both cytokines was much less from human bronchial epithelial cells (iHBEC) but levels were also affected by capsule. Pneumolysin stimulates CXCL8 release from both cell lines. Suppression of CXCL8 homologue (CXCL2/MIP-2) release by the capsule was also observed *in vivo* during intranasal colonization of mice but was only discernable in the absence of pneumolysin.

When pneumococci were administered intranasally to mice in a model of long term, stable nasopharyngeal carriage, encapsulated *S. pneumoniae* remained in the nasopharynx whereas the nonencapsulated pneumococci disseminated into the lungs.

Pneumococcal capsule plays a role not only in protection from phagocytosis but also in modulation of the pro-inflammatory immune response in the respiratory tract.

## Introduction

Two of the main virulence factors of *Streptococcus pneumoniae* are the polysaccharide capsule that surrounds most *S. pneumoniae* strains and the toxin pneumolysin [1]. It has been shown that pneumolysin can stimulate the innate immune response including release of the inflammatory cytokine CXCL8 from the host's airway epithelial cells [2]–[4].

The pneumococcal capsule is mainly composed of polysaccharides, with each capsule type having a different composition and linkage of the sugars and other components [5]. *S. pneumoniae* is classified into over 90 different serotypes on the basis of antibody reactions with the capsule [6]–[9]. Some serotypes frequently colonize the human nasopharynx asymptomatically whereas others are more associated with invasive diseases such as pneumonia, sepsis or meningitis, but are found less frequently in the nasopharynx because they colonize for a shorter duration [10]–[12].

Epithelial cells express pattern-recognition receptors (PRRs) that are required to signal the presence of pathogens and to recruit and activate professional antigen presenting cells such as macrophages or dendritic cells [13]. Numerous pro-inflammatory chemokines and cytokines are secreted such as CXCL8, IL-6, IL-1β, granulocyte-macrophage colony stimulating factor (GM-CSF), transforming growth factor (TGF) α and –β [14]. Secretion of cytokines tends to be a brief, self-limited event with synthesis beginning with gene transcription and mRNA having a short half-life [15].

Toll-like receptors (TLRs) 2-6 are expressed on airway epithelial cells. TLR2 is the principal receptor for recognition of bacterial components (e.g. lipoprotein, lipoteichoic acid, peptidoglycan, GPI anchor) and some viral envelope proteins [16]. TLR signaling in epithelial cells is not only important for microbial defence but also for mucosal homeostasis which is determined by the magnitude of signaling [13]. CXCL8 plays a major role in the initial control of respiratory tract infection due to its chemotactic activity for neutrophils and monocytes [17] and can be secreted by all cells which have TLRs [18].

In the current study we tested the role of the pneumococcal capsule in pro-inflammatory cytokine induction using human pharyngeal and bronchial epithelial cells and in a murine model of nasopharyngeal colonization. We also looked at the effect of the capsule on the ability of the bacteria to disseminate into the lungs following nasopharyngeal colonization.

## Materials and Methods

### Ethics statement

All animal experiments were performed at the University of Liverpool and with prior approval from the UK Home Office and the University Ethics Committee.

### Bacteria

The bacterial wild type and mutants strains used are listed in Table 1. The capsule mutants were constructed according to the protocols described previously [9], [19]. The pneumolysin mutant was a kind gift from Professor Jeremy Brown (University College London, UK) [20]. For the construction of D39*cps^-^ply^−^* mutant, the D39*cps^−^* mutant was used and mutant construction performed according to the method described previously [21]. Briefly, the up- and downstream flanking regions of the pneumolysin-gene were amplified using iProof polymerase (Bio-Rad, Switzerland) using the following primers: upstream forward primer KO_Ply_us_F 5′-GATTGATAATACCAGCACTC-3′, upstream reverse primer KO_Ply_us_R 5′-GGTAGAGGATAAGGTAG-3′, downstream forward primer KO_Ply_ds_F 5′-ATCGTAATTCATAGCTAG-3′ and downstream reverse primer KO_Ply_ds_R. PCR conditions were: 98°C for 30 sec, 35 cycles of 98°C for 10 sec, 55°C for 15 sec, 72°C for 20 sec and then 72°C for 10 min. PCR, using the same conditions, was also performed to amplify a spectinomycin cassette from the plasmid. The primers (us_Ply_Spec_F1 5′-CTAGCTATGAATTACGACTAGTGGATCCCCCGTTTGA-3′ and Spec_ds_Ply_R1 5′-CTACCTTATCCTCTACCATAGTTCCCTTCAAGAGCGATACC-3′) were designed to create overhangs which allowed fusion of the three PCR products as described elsewhere [22]. The fusion reaction, using Phusion High Fidelity Polymerase (Fisher Scientific, Switzerland), was: 98°C for 1 min, 10 cycles of 98°C for 10 sec, 50°C for 15 sec, 72°C for 1 min then the primers (KO_Ply_us_F and KO_Ply_ds_R) were added followed by 25 cycles of 98°C for 10 sec, 62°C for 15 sec, 72°C for 2 min 30 sec and then 72°C for 10 min. The amplified construct was then isolated from a 1% agarose gel. After transformation, clones were selected on CSBA plates supplemented with 200 μg/ml spectinomycin [23]. Incubation was performed under anaerobic conditions. Knockout of the pneumolysin gene was confirmed by PCR and by sequencing using primers KO_Ply_us_F and KO_Ply_ds_R using the following conditions: 98°C for 30 sec, 35 cycles of 98°C for 10 sec, 55°C for 15 sec, 72°C for 1 min 15 sec followed by 72°C for 10 min [24].

**Table 1:** 

Strain	Description	Capsule	Pneumolysin
D39	Wild type serotype 2 (NCTC 7466)	+	+
D39*cps^−^*	Mutant lacking capsule [33]	−	+
D39*ply^−^*	Mutant lacking pneumolysin [20]	+	−
D39*cps^−^ply^−^*	Mutant lacking both capsule and pneumolysin (current study)	−	−
110.58	Wild type nonencapsulated [34]	−	+
110.58::D39*cps*	Mutant with serotype 2 capsule [19]	+	+

Bacteria were plated on Columbia sheep blood agar (CSBA) plates and incubated overnight at 37°C and 5% CO_2_. Liquid cultures of bacteria were prepared using either 5 ml of Chemically Defined Medium (CDM) [25] supplemented with 50 mM of filter-sterilized sucrose or 5 ml of Brain Heart Infusion (BHI) broth. Bacteria were grown to mid-log phase, meaning to OD_600_ of 0.1 to 0.2 in CDM and to OD_600 nm_ of 0.5 to 0.8 in BHI, and then counted in a Neubauer chamber. The bacteria were pelleted, washed twice with pyrogen-free PBS then resuspended in 1 ml Eagle's minimum essential medium (MEM; Invitrogen, Basel, Switzerland) without FCS and warmed in a water bath to 37°C.

### Epithelial cell culture, exposure to pneumococcus and cytokine assays

The human pharyngeal epithelial cell line Detroit 562 (ATCC CCL 138) was cultured as published earlier in MEM supplemented with 10% of heat-inactivated fetal calf serum (FCS), 2 mM of L-glutamine (Invitrogen, Basel, Switzerland), 1% sodium bicarbonate (Invitrogen, Basel, Switzerland), 1× MEM non-essential amino acid solution (Sigma, St. Louis, MO, USA), 1 mM sodium pyruvate (Sigma, St. Louis, MO, USA), 100 μg/ml streptomycin and 100 U/ml penicillin at 37°C in 5% CO_2_ [26]. Cells were grown in 24-well plates to a confluent cell layer (≈3×10^5^ cells per well). MEM containing the bacteria at three CFU concentrations (1, 1.5 and 2 × 10^6^) were added to the Detroit 562 cells and the plates centrifuged at 173 g for 5 min at 25°C. After 24 h of incubation at 37°C and 5% CO_2_ supernatants from the wells were collected in Eppendorf tubes, spun down at 132 g for 3 min at room temperature, and then stored at -80°C until further use.

Immortalised human bronchial epithelial cells (iHBEC) were kindly provided by Professor Jerry W. Shay, (University of Texas, Dallas, USA) [27]. The iHBECs were grown in Keratinocyte serum-free media (Invitrogen) supplemented with epidermal growth factor and bovine pituitary extract. All cells were grown and experimented upon in humidified 5% CO_2_, 95% humidity air at 37°C, in the absence of antibiotics. Confluent iHBECs monolayers were grown in 24 well plates and incubated in serum-free media for 18 h prior to investigation. Unstimulated cells were used as controls. Cells were cultured in 24 well cell culture plates for 24 h with medium or live *S. pneumoniae*. After incubation, culture supernatants were collected, centrifuged at 10 000 g for 5 minutes to remove cellular debris and filter sterilized and stored at −80°C until assayed.

The amounts of CXCL8 and IL-6 were measured by ELISA (R&D systems ELISA kits, Abingdon, United Kingdom).

The experiments with both of the epithelial cell lines were performed in triplicate on three different days at three CFU concentrations (1, 1.5 and 2 × 10^6^) in each experiment.

### Mouse model

Age-matched 8–12 week old female MF1 mice (Charles River, UK) were intranasally infected with 1 × 10^5^ colony forming units of *S. pneumoniae* in 10 μl PBS as previously described [28]. Mice were sacrificed at pre-determined time points post-infection and organs removed for assessment of bacterial numbers and ELISA analysis. Nasopharynx and lungs were homogenized in PBS and serially diluted onto blood agar for enumeration of bacterial numbers by the Miles and Misra method. For the CFU counting, gentamicin plates were used to select for pneumococci and in the case of the bacteria that disseminated to the lung, colonies were picked and streaked onto plates with an optochin disc to confirm that they were pneumococci. Homogenates were retained for use in ELISA to measure a murine homologue of human CXCL8, CXCL2/MIP-2, using a kit from R&D systems.

### Statistics

Student *t* test was used to assess the significance of the results.

## Results and Discussion

We investigated the effect of capsule on CXCL8 and IL-6 induction in cells of the upper and lower respiratory tract using wild type *S. pneumoniae* serotype 2 (strain D39) and its capsule-deleted mutant. In addition, we compared the naturally occurring nonencapsulated clinical isolate 110.58 with its mutant in which the capsule of D39 has been inserted. Furthermore, D39 mutants in which pneumolysin alone was deleted or both pneumolysin and capsule were deleted were tested to investigate the role of capsule in the presence or absence of toxin (see Table 1 for bacterial wild type and mutant strains).

**Figure 1:**
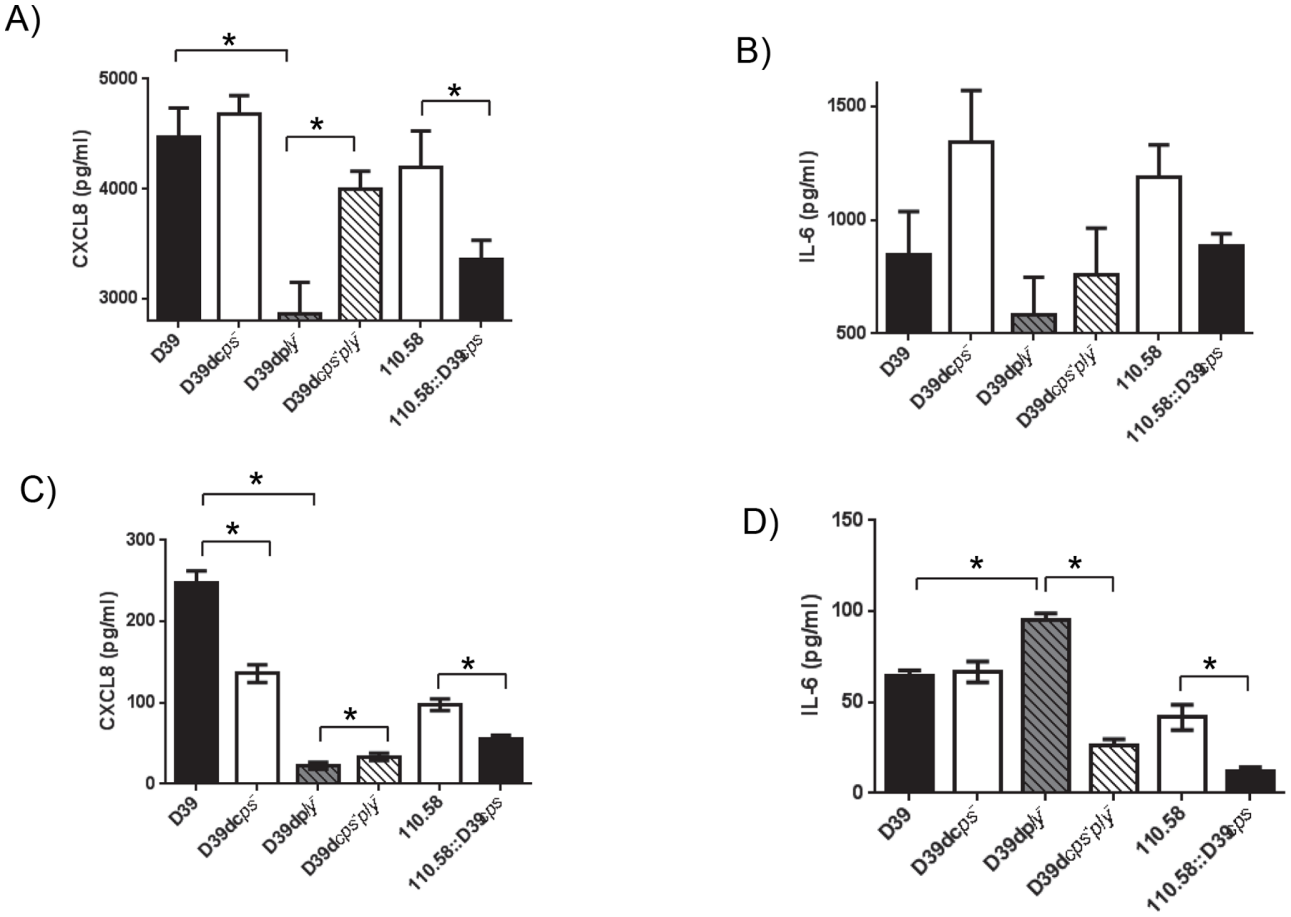
Effect of capsule and pneumolysin on CXCL8 and IL-6 induction in human nasopharyngeal and bronchial epithelial cells. Detroit 562 nasopharyngeal epithelial cells (A and B) and bronchial epithelial cells (C and D) were assessed for CXCL8 (A and C) and IL-6 (B and D) release after exposure to wild type or mutant pneumococcal strains. All experiments were performed in triplicate at each of three CFU concentrations (1, 1.5 and 2 × 10^6^) and the results pooled for each strain. Note different scales of Y axes. Error bars indicate SEM. * indicates significant difference.

Deletion of capsule from D39 caused a small, but non-significant, increase of CXCL8 from the upper respiratory tract cells and insertion of the D39 capsule into strain 110.58 significantly decreased CXCL8 levels (p  =  0.04) (Figure 1A). Deletion of pneumolysin significantly decreased CXCL8 compared to wild type control (p  =  0.0009) but CXCL8 levels increased when the capsule was additionally deleted when compared to pneumolysin deletion alone (p  =  0.0036). The same pattern was seen for IL-6 but the cytokine concentrations were lower and not significantly different between cells exposed to different strains (Figure 1B). For the bronchial epithelial cells, deletion of capsule in D39 caused a decrease in CXCL8 (p < 0.0001) but insertion of D39 capsule in strain 110.58 also caused a decrease in CXCL8 (p < 0.0001) (Figure 1C). These responses were in contrast to upper respiratory cells indicating a clear niche difference in host responses to pneumococcal capsule with the caveat that these are *in vitro* findings and we cannot conclude whether the pattern of cytokine release would be the same from the epithelial cells in their original anatomical locations. Deletion of pneumolysin in D39 decreased CXCL8 levels in keeping with the response of upper respiratory cells (p < 0.0001), however CXCL8 was only slightly increased upon the additional deletion of the capsule (p < 0.0001) once again indicating a capsule-dependent difference in upper versus lower respiratory tract responses. IL-6 values were low with no difference between D39 and its capsule-deficient mutant but with levels reduced by insertion of the capsule into strain 110.58 (p  =  0.0002). Deletion of pneumolysin increased IL-6 level (p < 0.0001) but additionally deleting the capsule reduced the level (p < 0.0001) (Figure 1D).

Overall, capsule restricted the release of CXCL8 from respiratory tract epithelial cells in contrast to pneumolysin, which stimulated the release of CXCL8.

No significant difference in cytotoxicity or haemolytic activity was observed between the encapsulated or nonencapsulated pneumococci (data not shown).

**Figure 2:**
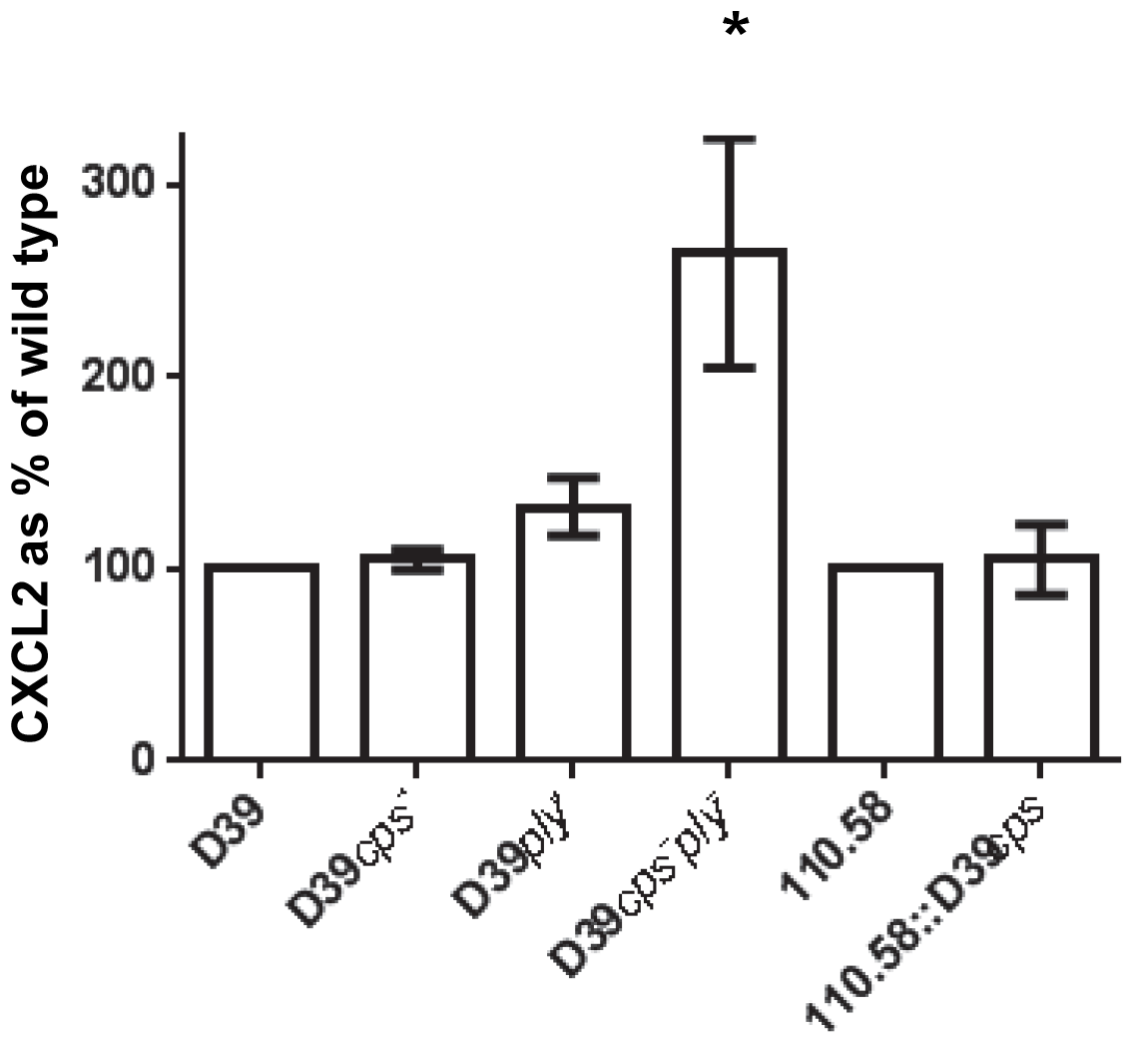
Effect of capsule and pneumolysin on CXCL8 homologue induction in the mouse nasopharynx. CXCL8 homologue (CXCL2/MIP-2) detected in nasopharyngeal homogenate of mice three days after exposure to wild type or mutant pneumococci expressed as a percentage of the value obtained with the wild type strain. Error bars indicate SEM. * indicates significant difference from value of the parent strain.

Having found that the capsule plays an important role in regulating CXCL8 induction *in vitro*, we next tested the influence of the capsule in a mouse model of nasopharyngeal carriage. Figure 2 shows that no difference in CXCL8 homologue (CXCL2/MIP-2) levels were detected between encapsulated or nonencapsulated strains in the nasopharynx and that deletion of pneumolysin did not have a measurable effect either. Importantly however, when the capsule was additionally deleted in the pneumolysin mutant, CXCL8 homologue level increased (p  =  0.05) supporting the *in vitro* finding that capsule suppresses CXCL8 release but that this effect may be masked by the presence of pneumolysin.

**Figure 3:**
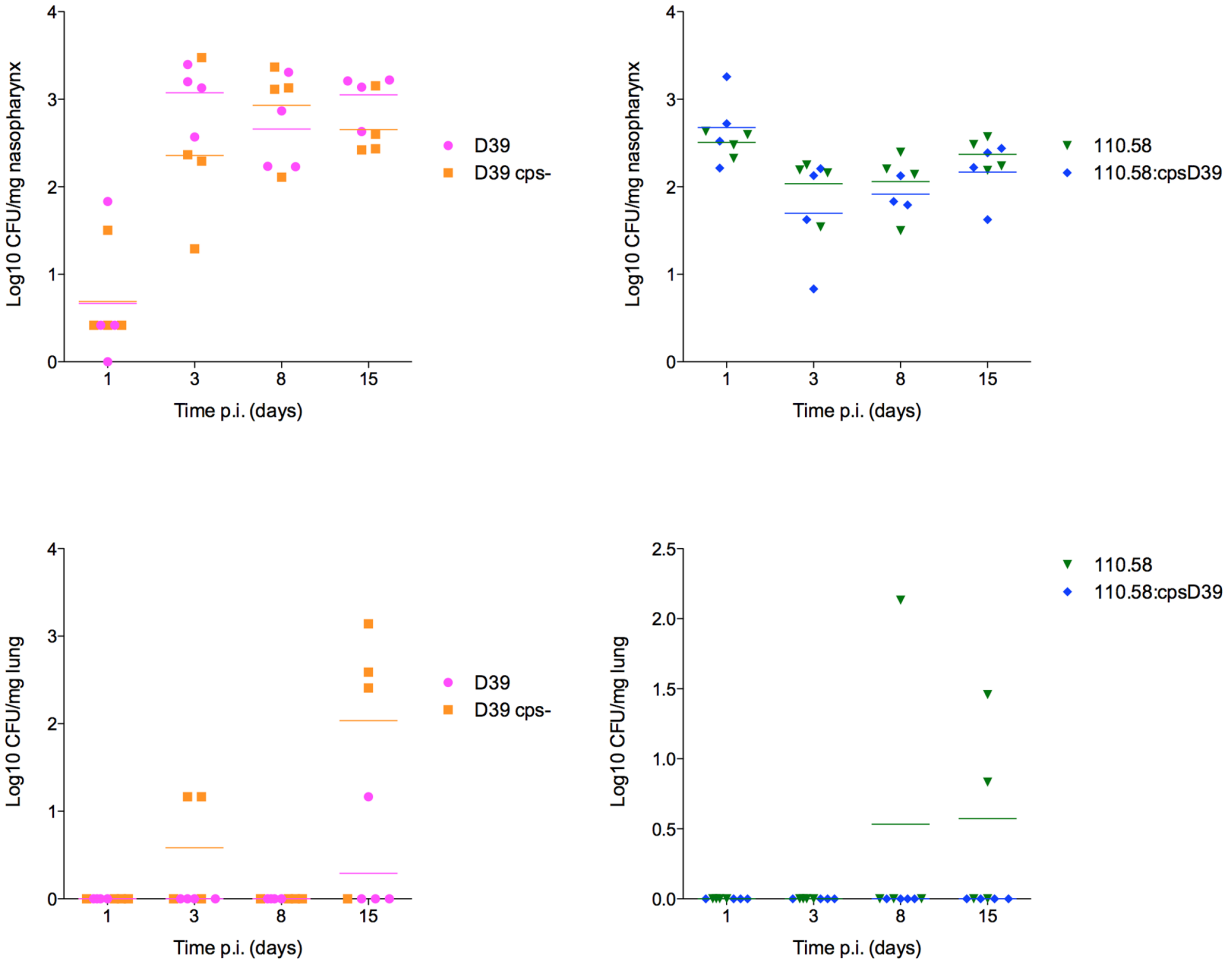
Capsule did not affect colonization of the nasopharynx but only nonencapsulated strains reached the lungs. Each symbol represents the CFU from the nasopharynx or lungs of an individual mouse on days 1, 3, 8 and 15 after intranasal inoculation. (No bacteria were detected at day 0 before any bacteria were administered.) Horizontal bars indicate means.

Interestingly, *in vivo* bacterial kinetics showed that, while the presence or absence of capsule did not appear to affect the ability of the pneumococcus to colonize the nasopharynx, only nonencapsulated pneumococci disseminated from the nasopharynx to the lungs (Figure 3) suggesting that the absence of capsule is important in facilitating pneumococcal movement from the nasopharynx to the lungs. Although nonencapsulated strains are expected to adhere to epithelial cells more efficiently than encapsulated strains, we did not detect higher numbers of nonencapsulated strains than encapsulated colonizing the nasopharynx.

The capsule is an important virulence factor as the thick polysaccharide layer helps the bacteria to escape opsonization and phagocytosis [29]. Encapsulation of the pneumococcus protects from complement activation. IgG and c-Adenosyl-monophosphate receptor protein (CRP) binding to the bacterial surface are reduced and thus activation of the classical pathway is impaired. Additionally, the degradation of C3b to iC3b is decreased by the capsule and phagocytosis by Fcγ receptor occurs less frequently [30]. Therefore, innate immunity initiated by encapsulated bacteria that causes macrophages and neutrophils to enter the nasopharynx as a result of chemotactic activity of CXCL8 will then not lead to efficient opsonophagocytosis of the bacteria due to the polysaccharide capsule. Here we suggest that restriction of the initial step of CXCL8 release from the epithelial cells by the polysaccharide capsule may also contribute to bacterial survival.

There has been a recent study which also investigated the innate immune response due to *Streptococcus pneumoniae* in epithelial cells and which did not find a clear difference between wildtype strains and their capsule knock out mutants in terms of CXCL8 induction [31]. However, this group used microarrays to characterize the *in vitro* transcriptional response whereas here we have detected CXCL8 itself both *in vitro* and in a mouse model of nasopharyngeal colonization. Another group, like us, found greater CXCL8 production in response to nonencapsulated pneumococci than their encapsulated parent strains [32].

In conclusion, we find that the pneumococcal capsule plays an important role in regulation of innate immunity by reducing CXCL8 release from upper respiratory tract cells and also by restricting pneumococcal dissemination into the lower respiratory tract, where the pneumococcus would normal elicit a strong pro-inflammatory response leading to its clearance. We would argue that this is in keeping with the natural role of the pneumococcus as a commensal of the upper respiratory tract, whereby its primary function is to establish colonization with limited or no host inflammation to sustain its longer term survival in the nasopharynx without host mediated clearance.
